# Supplementary material for: Over-Winter Survival and Nest Site Selection of the West-European Hedgehog (Erinaceus europaeus) in Arable Dominated Landscapes
Source: Animals (Basel). 2020 Aug 19;10(9):1449. doi: 10.3390/ani10091449 (PMC7552789; doi:10.3390/ani10091449)
Supplement: Supplementary file 1 [file animals-10-01449-s001.zip › Lucy Bearman Brown Hibernation paper Supplementary Figures.docx]

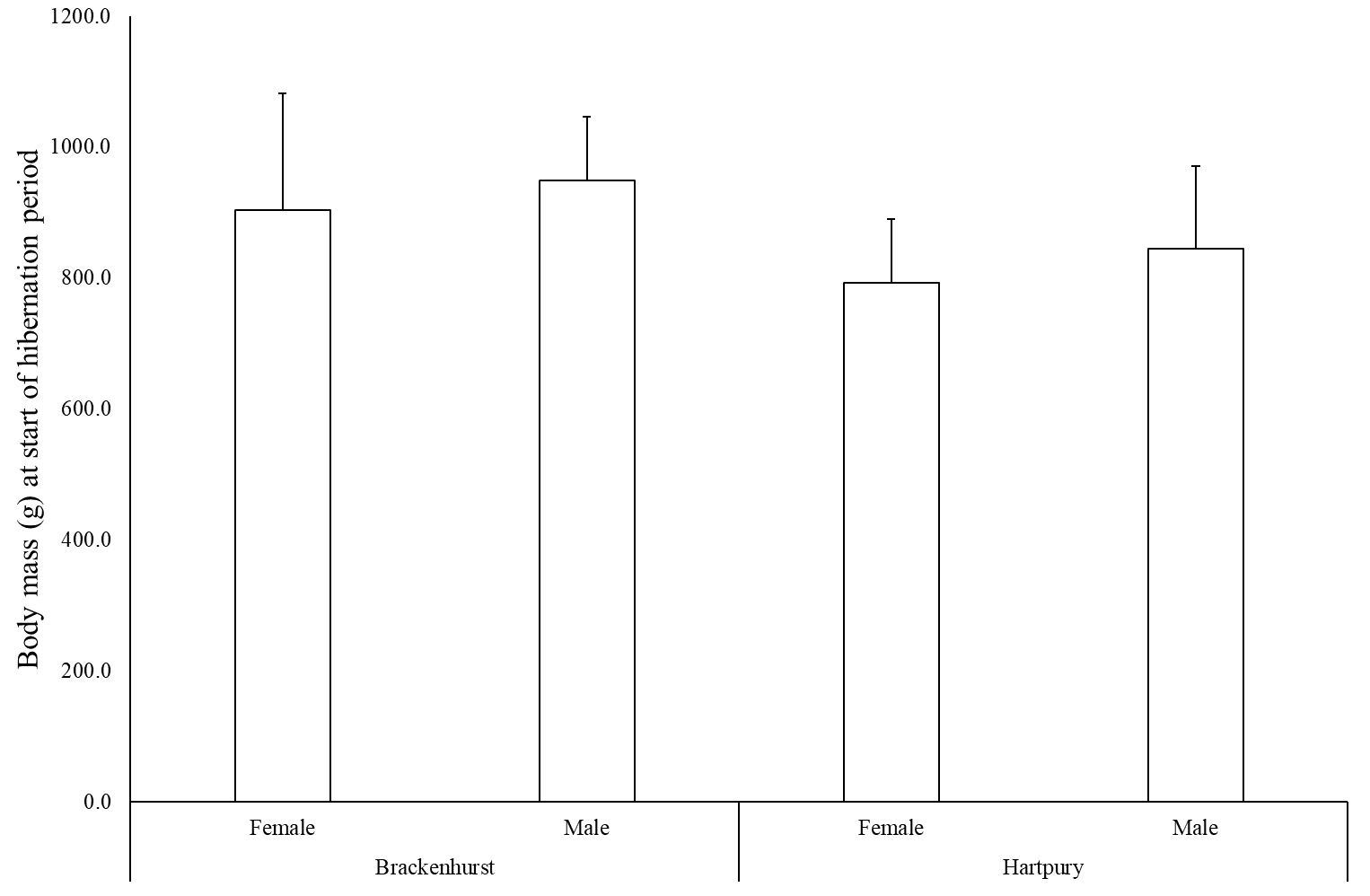


(a) Body mass at the start of hibernation

(b) Percentage mass change during hibernation


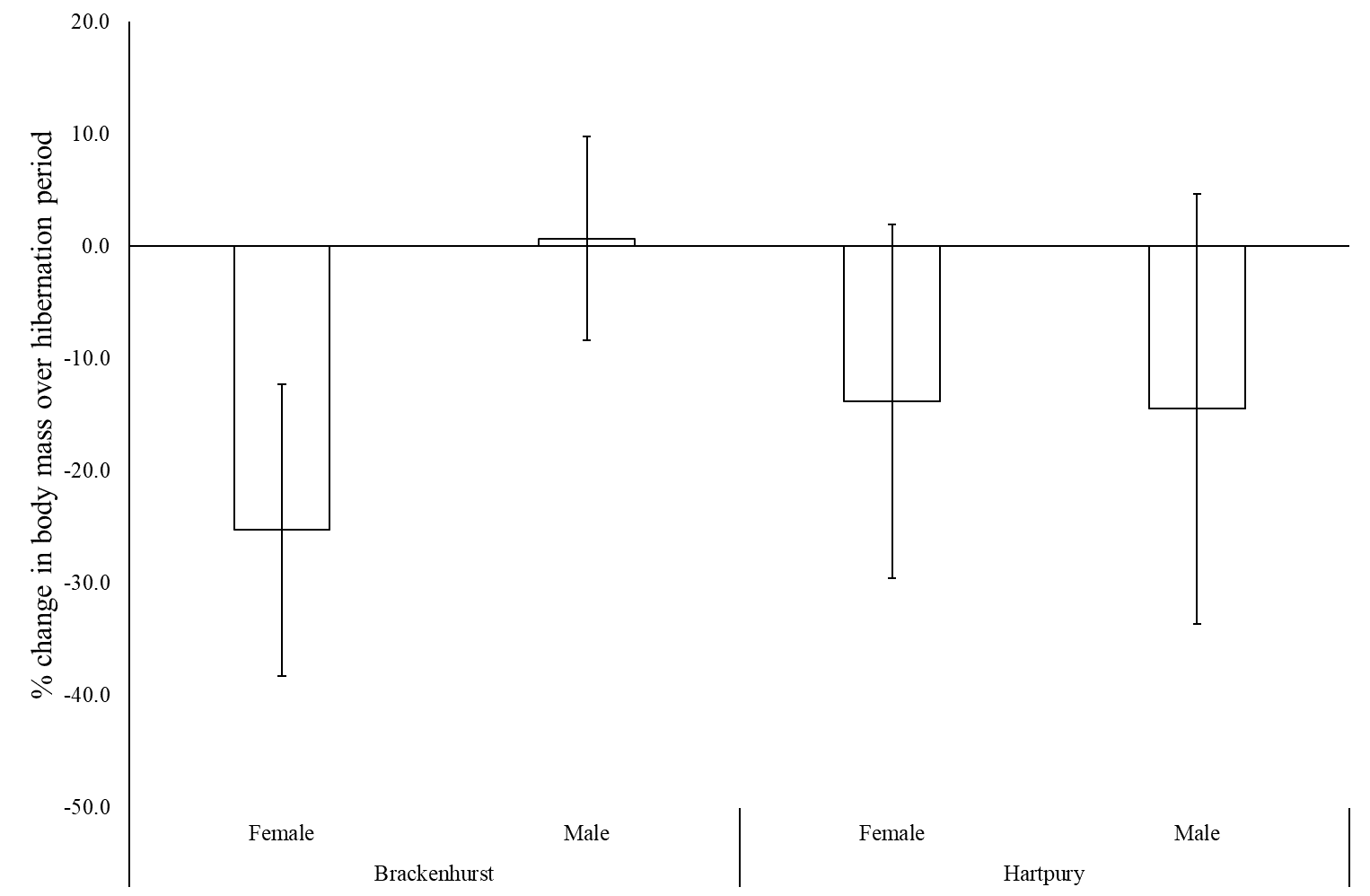


**Supplementary Figure 1.** Mean (±SD) (**a**) body mass (g) at the start of the hibernation season, and (**b**) percentage mass change during the hibernation period in relation to site and sex (Brackenhurst: n = 5♀:5♂; Hartpury: n = 6♀:5♂)
