# Supplementary material for: Over-Winter Survival and Nest Site Selection of the West-European Hedgehog (Erinaceus europaeus) in Arable Dominated Landscapes
Source: Animals (Basel). 2020 Aug 19;10(9):1449. doi: 10.3390/ani10091449 (PMC7552789; doi:10.3390/ani10091449)
Supplement: Supplementary file 1 [file animals-10-01449-s001.zip › Lucy Bearman Brown Hibernation paper Supplementary Tables v2.docx]

**Supplementary Table S1.** Summary of the dominant materials used in winter nest construction at Brackenhurst and Hartpury. Data for 2015-2016 and 2016-2017 combined. Figures in parentheses are the number of nests where the material was recorded as a secondary material. Sample sizes are less than the total number of nests used by study animals as not all nests were accessible.

| **Material** | **Brackenhurst** | **Hartpury** | **Total** |
| --- | --- | --- | --- |
| Broad leaves | 32 (49) | 42 (42) | 74 (91) |
| Conifer | 0 (5) | 0 (0) | 0 (5) |
| Grass | 12 (20) | 5 (9) | 17 (29) |
| Herbaceous plants | 1 (18) | 0 (1) | 1 (19) |
| Ivy | 7 (14) | 0 (0) | 7 (14) |
| Litter / plastic | 0 (0) | 0 (20) | 0 (20) |
| Moss | 1 (4) | 0 (1) | 1 (5) |
| Ornamental bush | 2 (7) | 0 (1) | 2 (8) |
| Ornamental grass | 2 (2) | 3 (2) | 5 (4) |
| Shredded garden waste | 2 (2) | 0 (0) | 2 (2) |
| Soil | 1 (1) | 0 (0) | 1 (1) |
| Stones | 1 (1) | 0 (0) | 1 (1) |
| Straw | 1 (3) | 0 (2) | 1 (5) |
| Twigs | 0 (3) | 0 (5) | 0 (8) |
| Unknown | 9 (1) | 32 (0) | 41 (1) |
| Total | 71 | 82 | 153 |

**Supplementary** **Table 2.** Cause of death (n = 9) from a sample of 31 individuals followed over two winter hibernation periods (2015-2016 or 2016-2017).

| **Cause** | **Brackenhurst** | **Hartpury** | **Total** |
| --- | --- | --- | --- |
| Badger predation | 0 | 3 | 3 (38%) |
| Road traffic | 0 | 2 | 2 (22%) |
| Natural causes | 0 | 1 | 1 (11%) |
| Euthanased^1^ | 0 | 1 | 1 (11%) |
| Unknown | 1 | 1 | 2 (22%) |
| Total | 1 | 8 | 9 |

^1^ Animal was euthanased by a veterinary surgeon because of a large facial tumour

**Supplementary Table 3.** General linear models comparing site and sex differences in (**a**) body mass (g) at the start of the hibernation season, and (**b**) absolute and (**c**) percentage mass change during the hibernation period (n = 21).

(a) Body mass at the start of the hibernation period

| **Variable** | **Degrees of freedom** | **Adjusted sum of squares** | **Adjusted mean sum of squares** | **F statistic** | **P** |
| --- | --- | --- | --- | --- | --- |
| SITE | 1 | 60668 | 60668.0 | 3.75 | 0.069 |
| SEX | 1 | 12647 | 12646.5 | 0.78 | 0.389 |
| SITE*SEX | 1 | 85 | 84.9 | 0.01 | 0.943 |
| Error | 17 | 274767 | 1612.7 |  |  |
| Total | 20 | 351287 |  |  |  |

(b) Absolute mass change during hibernation

| **Variable** | **Degrees of freedom** | **Adjusted sum of squares** | **Adjusted mean sum of squares** | **F statistic** | **P** |
| --- | --- | --- | --- | --- | --- |
| SITE | 1 | 170 | 169.5 | 0.01 | 0.919 |
| SEX | 1 | 83754 | 83754.2 | 5.21 | 0.036 |
| SITE*SEX | 1 | 74755 | 74755.3 | 4.65 | 0.046 |
| Error | 17 | 273349 | 16079.4 |  |  |
| Total | 20 | 425417 |  |  |  |

(c) Percentage mass change during hibernation

| **Variable** | **Degrees of freedom** | **Adjusted sum of squares** | **Adjusted mean sum of squares** | **F statistic** | **P** |
| --- | --- | --- | --- | --- | --- |
| SITE | 1 | 18.26 | 18.26 | 0.08 | 0.776 |
| SEX | 1 | 835.16 | 835.16 | 3.81 | 0.067 |
| SITE*SEX | 1 | 923.28 | 923.28 | 4.22 | 0.056 |
| Error | 17 | 3721.86 | 218.93 |  |  |
| Total | 20 | 5424.88 |  |  |  |
